# Association between pre-bronchoscopy time of illness and post-bronchoscopy discharge time in pediatric patients with foreign body aspiration: retrospective cohort study in a Peruvian referral center, 2014-2019

**DOI:** 10.17843/rpmesp.2023.404.12977

**Published:** 2023-12-18

**Authors:** Esteban Andrés Huarhua Jimenez, Alejandro Kruchinsky Lozada, Marcelo Galdos Bejar, Nilton Yhuri Carreazo

**Affiliations:** 1 Universidad Peruana de Ciencias Aplicadas, Lima, Peru. Universidad Peruana de Ciencias Aplicadas Universidad Peruana de Ciencias Aplicadas Lima Peru; 2 Pediatric Emergency Hospital, Lima, Peru. Pediatric Emergency Hospital, Lima, Peru Lima Peru

**Keywords:** Respiratory Aspiration, Airway Management, Pediatrics, Hospitalization, Peru

## Abstract

**Objective.:**

To determine the association between pre-bronchoscopy time of illness and post-bronchoscopy discharge time in pediatric patients with foreign body aspiration.

**Materials and methods.:**

Retrospective cohort study. Medical records were studied and reviewed at the Pediatric Emergency Hospital of Lima, Peru. We reviewed 324 medical records, and selected 183 because they had the diagnosis of foreign body aspiration and complete data. Fisher’s exact test and Mann Whitney U test were used for the bivariate analysis, while Poisson regression was used to calculate the Relative Risk (RR) and its 95% confidence interval (CI).

**Results.:**

We included 183 patients, of whom 65.6% were men with a mean age of 2.4 years. The most frequent location of the foreign body was the right bronchial tree and most of them were made of organic material. The majority (72.7%) of patients were discharged before 24 hours. We found an association between the time of illness prior to bronchoscopy and post-bronchoscopy discharge time (RR: 2.94, 95%CI: 1.72 - 5.01).

**Conclusions.:**

The time of illness prior to bronchoscopy and the length of hospital stay after removal of the foreign body were significantly associated when adjusted for age, sex, type of foreign body and mouth sweep maneuver as a rescue measure. Our findings are relevant because they highlight the importance of early care, timely diagnosis and early management of pediatric patients.

## INTRODUCTION

Foreign body ingestion or aspiration is one of the most common emergencies in pediatric patients ^1^. Severity depends on factors such as the type of the object: organic such as insects or seeds or inorganic such as toys, metal objects, plastics, etc., as well as the location and the time from aspiration to removal of the foreign body ^2^. Foreign body aspiration is a frequent cause of morbidity and mortality in children, and it was the cause of 160 deaths and more than 17 thousand emergency department visits in children under 14 years of age in the United States during the year 2000 ^3^. In Spain, the incidence rate peaks at the first two years of life ^4^. A study in Brazil reported that 50% of cases occur between the first and third year of life, mostly in males ^5^. Furthermore, this same study showed that most children under three years of age aspirated organic foreign bodies (86%) while those older than three years of age aspirated inorganic foreign bodies more frequently (75%) ^5^. Similarly, a study in Switzerland reported that 68.3% of patients were between 1 and 3 years old, and 60% presented symptoms in the last 24 hours ^6^.

The time of removal of the foreign body obstructing the patient’s airway is one of the most important factors to be evaluated ^2^^,^^7^. An article carried out in China ^7^ described a correlation between the retention time of the foreign body in the airway and the length of hospital stay (r= 0.189; p<0.001), which implies that the longer the removal time, the longer the hospital stay. However, a study by Hidaka *et al.* showed that there is no association between the time interval from the aspiration episode to the extraction bronchoscopy and a prolonged hospital stay ^8^.

In Peru, foreign bodies in tracheal and bronchial airways can be extracted by fibrobronchoscopy (flexible bronchoscopy) or rigid bronchoscopy, performed by a physician with experience in pediatric airway management ^9^ and familiar with pediatric endoscopic equipment. However, the scarcity of human resources (pediatric pulmonologist), materials and infrastructure; added to the characteristics of our referral system ^10^ represent important limitations in the care of this condition. This medical emergency requires specialized, timely and effective management, since the presence of complications increases as long as the foreign body remains in the airways ^2^^,^^9^. Therefore, this study aimed to determine the association between the time of illness prior to extraction and post-extraction hospital stay.

**KEY MESSAGES**

Motivation for the study: Foreign body aspiration in pediatric patients is an emergency, and its possible complications can be avoided by timely and adequate management.

Main findings: Patients with more than 48 hours between foreign body aspiration and bronchoscopy have approximately three times the risk of late discharge from the hospital. The most frequent location was the right bronchial tree, with predominance of male sex, organic material as foreign body and mean age of 2.4 years.

Implications: This study allows us to recognize the importance of promoting timely care and diagnosis, allowing early management of pediatric patients with foreign body aspiration.

## MATERIALS AND METHODS

### Study design and population

This was a retrospective cohort study. The study population were pediatric patients of the Pediatric Emergency Hospital (HEP) of Lima, Peru. We considered everyone younger than 18 years of age according to the definition of pediatric population by the World Health Organization (WHO) ^11^.

The HEP has been recognized since 2005 as a category III-1 healthcare center and is one of the main centers specialized in the management of pediatric emergencies and urgencies, such as the removal of foreign bodies from the airway ^12^.

### Inclusion criteria

We considered all patients between 0 and 18 years old, who were discharged with the diagnosis code T17 (foreign body in airway) from the International Classification of Diseases 10th (ICD-10), who underwent bronchoscopy (rigid or flexible) and were attended between 2014 and 2019 in the HEP.

### Exclusion criteria

Patients with a foreign body located in places other than the larynx, trachea or right or left bronchial tree and those in which the time of illness was longer than 18 days were excluded, considering that longer times would not be pediatric emergencies ^13^. Patients with absence of foreign bodies and medical records that did not comply with adequate filling were also excluded.

### Sample size calculation

We used the article by Tomaske *et al*. ^6^ to calculate the sample size. The aforementioned study found that patients who had a time of illness greater than 24 hours (exposed) had a risk of late discharge of 40% and the risk of those who had a time of illness less than 24 hours (unexposed) was 29.1%. We considered the unexposed/exposed ratio of 0.60. With these values, we calculated the sample size in the EPIDAT ® version 4.2 program (Xunta de Galicia, GAL, ESP), obtaining an initial sample size of 183 patients.

### Data Collection

Data were obtained by reviewing the medical records of pediatric patients at the HEP. Data collection was performed by requesting permission from the HEP Research and Teaching area. We requested all records that included patients with diagnosis, management and discharge of the patient, in order to identify all patients with a diagnosis of foreign body in the airway. Data collection was performed by two authors (EAHJ and AKL) using a data collection form, which was used to extract all variables included in the study.

### Variables

The time of illness prior to bronchoscopy, defined as the time elapsed since the aspiration of the foreign body, was considered as the independent variable. This variable was categorized into two groups (0 to ≤2 days and >2 days) ^14^. The dependent variable was post-bronchoscopy discharge time, defined as the time elapsed from foreign body removal to patient discharge, and was categorized as early discharge, when it occurred before or at 24 hours, and as late discharge, when it occurred after 24 hours ^15^.

The covariables were sociodemographic characteristics such as sex (male or female), age (years), origin (Lima or province), previous hospitalization and type of patient (direct or referred). In addition, we considered clinical findings (cough, wheezing, decreased vesicular murmur, abnormal findings on chest X-ray), and rescue maneuvers outside the health facility (mouth sweep, Heimlich maneuver and tracheotomy) ^10^. Among the characteristics of the bronchoscopy, we considered the location of the foreign body (trachea, right bronchial tree, left bronchial tree or larynx) ^6^, the type of foreign body (organic and inorganic) ^10^, the type of bronchoscopy performed for diagnosis (rigid, flexible or both) and extraction (rigid or flexible), as well as the duration of bronchoscopy in minutes (numerical) and the presence of complications (pneumonia, atelectasis, pneumothorax) ^10^.

### Statistical Analysis

We prepared a data base by double digitization in Microsoft Excel 2019 ® (Microsoft Corporation, CA, USA) which was exported to the statistical package STATA 16 ® (Stata Corporation, College Station, Texas, USA). The database was cleaned and incomplete medical records were eliminated for the main and secondary variables of interest.

For the descriptive analysis, we evaluated the numerical variables using the Shapiro Wilk test to verify whether or not they presented a normal distribution. Thus, we proceeded to describe the numerical variables: age and duration of extraction bronchoscopy using medians and interquartile ranges (IQR) because neither of the two variables had normal distribution. Frequencies and percentages were used to describe the categorical variables. Fisher’s exact test was used during the bivariate analysis to determine the difference between the proportions of the categorical variables and the dependent variable, while the Mann-Whitney U test was used for the numerical variables. A p<0.05 value was considered statistically significant.

The Poisson regression model was used during the multivariate analysis to calculate the relative risk (RR) and their respective 95% confidence intervals (CI). We calculated a model adjusted for the following variables: age, gender, type of foreign body and mouth sweep as a rescue maneuver. This last variable was included by statistical criterion. The type of foreign body was considered as a possible confounding variable despite not obtaining a statistically significant value in our study, by virtue of its epidemiological criterion ^16^.

### Ethical Aspects

The study protocol was submitted to the Ethics Committee of the Universidad Peruana de Ciencias Aplicadas and the Teaching and Research Support Office of the Hospital de Emergencias Pediátricas (code FSC-CEI/55-10-20), and data collection only took place when approval was obtained from both entities. No interventions were performed on the study subjects, nor were patients’ personal identification data included. The clinical data collected for our study were entered into a database, to which only the principal researchers had access.

## RESULTS

**Figure 1:**
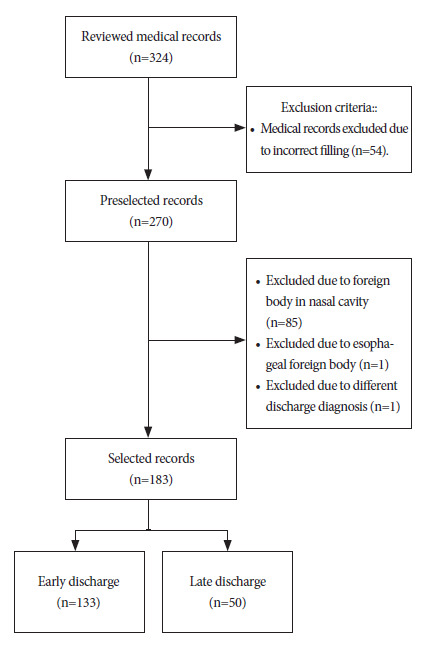
Participant selection flowchart.

We reviewed 324 medical records of pediatric patients diagnosed with foreign bodies in the airway. These patients underwent extraction by fibrobronchoscopy and/or rigid bronchoscopy at the HEP in the city of Lima, Peru. Of these 324 medical records, 141 were excluded and 183 histories were included in the study (Figure 1).

Of the total number of pediatric patients included in the study, we found that according to their sociodemographic characteristics, 120 (65.6%) patients were male, with a median age of 2.4 years (IQR: 1.3-7). Most patients (80.3%) attended HEP by referral (Table 1). The most frequent symptom, among the clinical and management variables of the patient with a diagnosis of foreign body, was cough (73.2%). Finally, the most frequently used rescue maneuver prior to extraction was sweeping the mouth with the finger (37.2%) (Table 1).

**Table 1:** Sociodemographic, clinical and intervention characteristics prior to management and treatment of pediatric patients diagnosed with airway foreign body (n=183 patients).

Characteristics	n	%
Sex		
Male	120	65.6
Female	63	34.4
Age (years)^a^	2,4	1.3-7
Origin		
Lima	65	35.5
Provinces	118	64.5
Type of patient		
Direct	36	19.7
Referred	147	80.3
Previous hospitalization		
Yes	33	18.0
No	150	82.0
Clinical findings		
Cough		
Yes	134	73.2
No	49	26.8
Wheezing		
Yes	53	29.0
No	130	71.0
Diminished vesicular murmur		
Yes	99	54.1
No	84	45.9
Chest X-ray		
Normal	66	35.7
Abnormal	117	64.3
Rescue maneuvers outside the facility		
Mouth sweep		
Yes	68	37.2
No	115	62.8
Heimlich maneuver		
Yes	5	2.7
No	178	97.3
Tracheotomy		
Yes	1	0.6
No	182	99.4

a Median and interquartile range.

According to the characteristics of the bronchoscopy performed on pediatric patients with a diagnosis of foreign body in the airways, we found that most patients had an illness time prior to bronchoscopy of less than or equal to two days (56.8%). Likewise, the most frequent location of the foreign body was the right bronchial tree (46.6%). The most frequent type of bronchoscopy was flexible bronchoscopy, as the initial technique (53.0%) and as the procedure that removed the foreign body (54.1%). The duration of bronchoscopy had a median of 20 minutes (IQR: 15-30) (Table 2).

**Table 2:** Bronchoscopy characteristics of pediatric patients (n=183 patients).

Characteristics	n	%
Time of illness prior to bronchoscopy		
0 to 2 days	104	56.8
> 2 days	79	43.2
Location of the foreign body		
Trachea	27	14.8
Right bronchial tree	78	42.6
Left bronchial tree	52	28.4
Larynx	26	14.2
Performed bronchoscopy		
Rigid bronchoscope	8	4.4
Flexible bronchoscope	97	53.0
Both	78	42.6
Extraction bronchoscope		
Rigid bronchoscope	84	45.9
Flexible bronchoscope	99	54.1
Duration of bronchoscopy (in minutes)^a^	20	15-30

a Median and interquartile range.

Regarding the characteristics of the pediatric patients after foreign body removal, most (72.7%) were discharged early (before or equal to 24 hours). The predominant type of foreign body in pediatric patients was organic material (59.6%). As for complications, the most frequent was pneumonia (24.6%), followed by atelectasis (18.0%) and pneumothorax (1.6%), this being the least frequent complication (Table 3).

**Table 3:** Characteristics of pediatric patients after foreign body removal (n=183 patients).

Characteristics	n	%
Post-bronchoscopy discharge time		
Early discharge (≤24 hours)	133	72.7
Late discharge (>24 hours)	50	27.3
Type of foreign body		
Organic	109	59.6
Inorganic	74	40.4
Complications		
Pneumonia		
Yes	45	24.6
No	138	75.4
Atelectasis		
Yes	33	18.0
No	150	82.0
Pneumothorax		
Yes	3	1.6
No	180	98.4

We found no differences between median patient age and post-extraction hospitalization time during bivariate analysis. Likewise, there were no differences between the proportions of previous hospitalization of patients with post-extraction hospitalization time. However, differences were found between the proportions of finger sweeping of the mouth as a rescue maneuver and post-extraction hospitalization time (Table 4). Additionally, differences were also found between the proportions of pre-bronchoscopy sick time and post-bronchoscopy discharge time (p<0.001). Finally, differences were found as well between the proportions of the occurrence of pneumonia and post-extraction hospitalization time (p<0.001) (Table 5).

**Table 4:** Contrast of sociodemographic and clinical variables with the length of time the patient remained in the health center after extraction (n=183 patients).

Characteristics	Early discharge (≤24 hours) (n = 133)		Late discharge (>24 hours) (n = 50)		p-value
	n	%	n	%	
Sex					
Male	90	75.0	30	25.0	0.384
Female	43	68.3	20	31.7	
Age (years)^a^	2,6	1.3-7.3	2,1	1.1-5.8	0.931^b^
Origin					
Lima	53	81.5	12	18.5	0.057
Provinces	80	67.8	38	32.2	
Type of patient					
Direct	28	77.8	8	22.2	0.534
Referred	105	71.4	42	28.6	
Previous hospitalization					
Yes	20	60.6	13	39.4	0.129
No	113	75.3	37	24.7	
Clinical findings					
Cough					
Yes	96	71.6	38	28.4	0.709
No	37	75.5	12	24.5	
Wheezing					
Yes	38	71.7	15	28.3	0.856
No	95	73.1	35	26.9	
Diminished vesicular murmur					
Yes	67	67.7	32	32.3	0.134
No	66	78.6	18	21.4	
Chest X-ray					
Normal	54	83.1	11	16.9	0.024
Anormal	78	66.7	39	33.3	
Rescue maneuvers outside the facility					
Mouth sweep					
Yes	56	82.0	12	17.7	0.026
No	77	67.0	38	33.0	
Heimlich maneuver					
Yes	2	40.0	3	60.0	0.127
No	131	73.6	47	26.4	
Tracheotomy					
Yes	0	0.0	1	100.0	0.273
No	133	73.1	49	26.9	

a Median and interquartile range; ^b^ p-value calculated using the Mann-Whitney U test.

**Table 5:** Contrast of bronchoscopy variables and pediatric patient complications with the length of hospital stay after foreign body removal (n=183 patients).

Characteristics	Early discharge (≤24 hours) (n = 133)		Late discharge (>24 hours) (n = 50)		p-value
	n	%	n	%	
Time of illness prior to bronchoscopy					
0 to 2 days	84	85.6	15	14.4	0.001
> 2 days	44	55.7	35	44.3	
Location of the foreign body					
Trachea	21	77.8	6	22.2	0.941
Right bronchial tree	56	71.8	22	28.2	
Left bronchial tree	37	71.2	15	28.9	
Larynx	19	73.1	7	26.9	
Type of foreign body					
Organic	77	70.6	32	29.4	0.502
Inorganic	56	75.7	18	24.3	
Performed bronchoscopy					
Rigid bronchoscope	8	100.0	0	0.0	0.124
Flexible bronchoscope	72	74.2	25	25.8	
Both	53	68.0	25	32.0	
Extraction bronchoscope					
Rigid bronchoscope	59	70.2	25	29.8	0.510
Flexible bronchoscope	74	74.8	25	25.2	
Duration of bronchoscopy (in minutes) ^a^	20	15-30	25	15-35	0.169 ^b^
Complications					
Pneumonia					
Yes	13	28.9	32	71.1	0.001
No	120	87.0	18	12.0	
Atelectasis					
Yes	19	57.6	14	42.4	0.050
No	114	76.0	38	240	
Pneumothorax					
Yes	2	66.7	1	33.3	1.000
No	131	72.8	49	27.3	

a Median and interquartile range; ^b^ p-value calculated using the Mann-Whitney U test.

During the multivariate analysis, the model adjusted for the variables age, sex, type of foreign body and mouth sweeping technique showed that the risk of having late discharge increases by 2.94 times (95% CI: 1.72-5.01; p=0.001) in those patients with more than 48 hours of sick time prior to extraction bronchoscopy (Table 6).

**Table 6:** Crude and adjusted regression analysis for hospitalization time greater than 24 hours post foreign body removal in pediatric patients at the Pediatric Emergency Hospital (n=183 patients).

Characteristics	Crude analysis		Adjusted analysis ^a^	
	RR (95% CI)	p-value	RR (95% CI)	p-value
Time of illness prior to bronchoscopy				
0 to 2 days	Ref.		Ref.	
>2 days	3.07 (1.81-5.22)	0.001	2.94 (1.72-5.01)	0.001

RR: relative risk; CI: confidence interval.

a Adjusted for age, gender, type of foreign body and mouth sweep.

## DISCUSSION

The most frequent location was in the right bronchial tree and the most frequent type of foreign body was organic materials among all 183 included patients. We found that 72.7% of patients were discharged before 24 hours. Our main finding was the association between the time of illness prior to bronchoscopy greater than two days and late discharge.

The moment of aspiration usually occurs while eating or playing, usually under adult supervision ^17^, which corresponds to the high exposure to new stimuli and foods at early ages, with organic objects being the most frequent ^18^, particularly food ^19^. This finding is consistent with another study by Bing Zhong *et al*. ^16^. Particularly, in our study, 59.6% of the foreign bodies removed were organic. This finding is relevant when considering that the type of foreign body is associated with the length of hospital stay ^8^, and that different types of foreign bodies can be associated with different types of injuries, for example, organic bodies are associated with upper respiratory tract injury ^20^.

Our study shows an association between the time of illness prior to bronchoscopy and the time of hospitalization after foreign body extraction. However, the study by Hidaka *et al*. ^8^, found no association between the variables of interest; they described that 74% of the cases underwent removal within two days of foreign body aspiration, while in our study only 53.8% of the cases underwent removal within two days of foreign body aspiration. This difference would indicate that in other contexts such as Japan, the diagnosis and removal of the foreign body is performed in less time compared to our context, thus reducing the risk of complications and hospitalization time. Thus, our findings could be explained by the problems of the medical referral system in Peru and the low availability of specialists ^10^. Likewise, the study by Hidaka *et al*. describes a low prevalence of foreign body aspiration in Japan ^8^, since they only found 77 cases of foreign body aspiration during the 23 years they evaluated (from 1988 to 2011). In contrast, our study considered a time interval of only six years (from 2014 to 2019), finding more than 183 cases. Therefore, these differences could lead to dissimilar results between the two studies.

However, several studies found an association between the time of illness and the time of post-extraction hospitalization ^7^^,^^21^. For example, a study conducted in China ^7^, reported a correlation between the time of foreign body retention in the airway and the time of hospitalization. Although this correlation was low (r=0.189), it was statistically significant (p<0.001). In our study, 56.8% of the participants had an illness time prior to bronchoscopy less than or equal to two days, a result similar to that reported in the study conducted in Chinese population, where 68.5% had a time of three days or less, which would mean that both studies found a relationship between both variables. However, it was not possible for the authors of the Chinese study to establish a cut-off point for the time of illness prior to bronchoscopy, as they considered this variable to be numerical. Additionally, another study performed in Spanish pediatric population showed a similar result to ours, where the authors report that there was a significant association between diagnostic delay and the appearance of long-term complications, without addressing intraoperative adverse events ^21^.

Prolonged illness prior to bronchoscopy doubles the risk of post-extraction hospitalization. Although asphyxia and cardiorespiratory arrest are more frequent shortly after aspiration, they can also occur in later days ^22^. Late diagnosis is frequently associated with atelectasis, lung abscesses and increased morbidity and mortality ^22^^-^^24^, due to inflammation occurring at the lower airway level ^16^. In fact, medical literature describes that foreign body aspiration causes inflammation in the airway, which prolongs pulmonary recovery time ^25^. The importance of this finding is evidenced in a study by Man Ki Chung *et al*. where they describe an association between inflammatory findings in the airway prior to foreign body removal and delayed pulmonary recovery ^25^. This fact can cause the need for extended hospitalization, which in turn, results in a higher risk of acquiring nosocomial infections ^26^.

The diagnostic or therapeutic delay can be explained by causes associated with the physician, resources, parents or uninformative medical records ^20^. Regarding the medical records, the condition may occur even without a history of choking or asphyxia in the pediatric population ^27^. For this reason, our results highlight the importance of timely management and correct evaluation of patients within the first 48 hours. As a complement, in order to ensure timely management and improve prognosis, it is recommended to train child caregivers, streamline referral systems and strengthen programs on the use of rigid and flexible bronchoscopy in pediatric patients. Therefore, in line with our findings, priority should be given to the availability of trained professionals and sufficient resources in health centers for the timely management of pediatric patients with foreign body aspiration, which would reduce complications and improve the prognosis of these patients.

This study has limitations. Patient evolution after discharge from HEP was not evaluated due to the nature of the study design. Although we only evaluated the data from the medical records, post-extraction complications are infrequent ^18^^,^^21^^,^^27^^,^^28^ and if they had occurred, the patient would have opted to return. Since the data were collected manually by means of a data collection form, information bias may have occurred. In addition, we did not include other variables relevant to this study, such as the activity that the patient was performing at the time of aspiration, since this information is usually not recorded in the medical records. Regarding external validity, since the data were obtained from a single hospital, our results cannot be extrapolated to different populations. However, it should be considered that HEP is one of the main referral institutions for pediatric patients in Lima, Peru, which allows us to determine the association of interest in an exploratory manner. Finally, there is no standardization of the main variables (time of discharge and time of illness prior to bronchoscopy). Nevertheless, our research uses cut-off points described in similar studies ^14^^,^^15^.

According to our results, most foreign body aspiration cases occurred in boys younger than three years old. In addition, patients with an illness time longer than two days between foreign body aspiration and bronchoscopy had 2.94 higher risk of a prolonged hospital stay (more than 24 hours) when adjusting for age, sex, type of foreign body and mouth sweep as a rescue maneuver.
