# Supplementary material for: Association between pre-bronchoscopy time of illness and post-bronchoscopy discharge time in pediatric patients with foreign body aspiration: retrospective cohort study in a Peruvian referral center, 2014-2019
Source: Rev Peru Med Exp Salud Publica. 2023 Dec 18;40(4):423–31. doi: 10.17843/rpmesp.2023.404.12977 (PMC11139497; doi:10.17843/rpmesp.2023.404.12977)
Supplement: Translation

# Translation: Asociación entre el tiempo de enfermedad previo a broncoscopia y tiempo de alta posbroncoscopia en pacientes pediátricos con aspiración de cuerpo extraño: estudio de cohorte retrospectivo en un centro de referencia peruano, 2014-2019

**Authors:** Esteban Andrés Huarhua Jimenez, Alejandro Kruchinsky Lozada, Marcelo Galdos Bejar, Nilton Yhuri Carreazo

**Affiliations:** 1 Universidad Peruana de Ciencias Aplicadas, Lima, Perú.; 2 Pediatric Emergency Hospital, Lima, Perú.

**Keywords:** Aspiración Respiratoria, Manejo de la Vía Aérea, Pediatría, Hospitalización, Perú

## Abstract

**Objetivos.:**

Determinar la asociación entre el tiempo de enfermedad previo a broncoscopia y el tiempo de alta posbroncoscopia en pacientes pediátricos con aspiración de cuerpo extraño.

**Materiales y métodos.:**

Estudio de cohorte retrospectivo. El estudio y la revisión de historias clínicas se realizó en el Hospital de Emergencias Pediátricas de Lima, Perú. Se revisaron 324 historias clínicas, de las cuales, 183 historias fueron seleccionadas por contar con diagnóstico de cuerpo extraño en vías aéreas y estar adecuadamente llenadas. Para el análisis bivariado se utilizó la prueba exacta de Fisher y U de Mann Whitney, mientras que para el cálculo del riesgo relativo (RR) y su respectivo intervalo de confianza (IC) al 95% se utilizó regresión de Poisson.

**Resultados.:**

Se incluyeron 183 pacientes, de los cuales, el 65,6% fueron hombres con una media de 2,4 años. La localización más frecuente fue el árbol bronquial derecho y cuerpo extraño de material orgánico. La mayoría (72,7%) de pacientes tuvieron un alta temprana antes de las 24 horas. Se encontró una asociación entre el tiempo de enfermedad previo a la broncoscopia y el tiempo alta posbroncoscopia (RR: 2,94, IC 95%: 1,72-5,01).

**Conclusiones.:**

Existe una asociación estadísticamente significativa entre el tiempo de enfermedad previo a la broncoscopia y el tiempo de estancia hospitalaria posextracción del cuerpo extraño al ajustar por edad, sexo tipo de cuerpo extraño y maniobra de boca como medida de rescate. Este hallazgo es relevante debido a que pone en evidencia la importancia de una atención temprana, diagnóstico oportuno y manejo precoz en el paciente pediátrico.

## INTRODUCCIÓN

La ingesta o aspiración de cuerpos extraños es una de las urgencias más comunes en pacientes pediátricos ^1^. La severidad depende de factores tales como el tipo: orgánico como insectos o semillas o inorgánico como juguetes, objetos metálicos, plásticos, etc., la localización y el tiempo desde que se aspiró hasta que se extrajo el cuerpo extraño ^2^. La aspiración de cuerpos extraños es una causa frecuente de morbimortalidad en niños y durante el año 2000, fue la causa de 160 muertes y más de 17 mil consultas en los servicios de urgencias en niños menores de 14 años en Estados Unidos ^3^. En España, se ha identificado un pico en la incidencia en los primeros dos años de vida ^4^. Un artículo realizado en Brasil reporta que el 50% de casos se presentan entre el primer y tercer año de vida, con predominancia del sexo masculino ^5^. Además, en este mismo estudio, la mayoría de los niños menores de tres años aspiraron un cuerpo extraño de tipo orgánico (86%) mientras que los mayores de tres años aspiraron un cuerpo extraño inorgánico con mayor frecuencia (75%) ^5^. De forma similar, en un estudio realizado en Suiza, el 68,3% de pacientes tenía entre 1 y 3 años, y el 60% presentó síntomas en las últimas 24 horas ^6^.

El tiempo de extracción del cuerpo extraño que obstruye la vía aérea del paciente es uno de los factores más importantes a evaluar ^2^^,^^7^. En un artículo realizado en China ^7^, se describe que existe una correlación entre el tiempo de retención del cuerpo extraño en vías aéreas y el tiempo de estancia hospitalaria (r= 0,189; p<0,001), lo que implica que a medida que el tiempo de extracción se prolonga la estancia hospitalaria se incrementa. Sin embargo, en un estudio realizado por Hidaka *et al.* mencionan que no existe asociación entre el intervalo de tiempo desde el episodio de aspiración hasta la broncoscopia de extracción y un tiempo prolongado de estancia hospitalaria ^8^.

En nuestro país, la extracción del cuerpo extraño en vías aéreas traqueales y bronquiales puede ser mediante la fibrobroncoscopia (broncoscopia flexible) o la broncoscopia rígida, realizada por un médico con experiencia en el manejo de la vía aérea pediátrica ^9^ y familiarizado con el equipamiento endoscópico pediátrico. Sin embargo, la escasez del recurso humano (neumólogo pediatra), material e infraestructura; sumado a las características de nuestro sistema de referencia ^10^ representan limitaciones importantes en la atención de esta patología. Esta urgencia médica supone un manejo especializado, oportuno y eficaz, debido a que la presencia de complicaciones aumenta a medida que el cuerpo permanece mayor tiempo en las vías aéreas ^2^^,^^9^. Por lo tanto, el objetivo del presente estudio fue determinar la asociación entre el tiempo de enfermedad previo a la extracción y la estancia hospitalaria posextracción.

**MENSAJE CLAVE**

Motivación para realizar el estudio: La aspiración de cuerpos extraños en pediatría es una emergencia, cuyas posibles complicaciones pueden evitarse mediante un manejo oportuno y adecuado.

Principales hallazgos: Aquellos pacientes con más de 48 horas entre la aspiración de cuerpo extraño y la broncoscopía presentan aproximadamente tres veces el riesgo de alta tardía. La localización más frecuente fue el árbol bronquial derecho, con predominancia de sexo masculino, material orgánico como cuerpo extraño y edad media de 2,4 años.

Implicancias: Este estudio permite reconocer la importancia de promover una atención y diagnóstico oportuno, que permita un manejo precoz del paciente pediátrico con aspiración de cuerpo extraño.

## MATERIALES Y MÉTODOS

### Diseño y población del estudio

El diseño de estudio fue una cohorte retrospectiva. La población de estudio fueron pacientes pediátricos del Hospital de Emergencias Pediátricas (HEP) de Lima, Perú. Para delimitar adecuadamente a la población pediátrica, se utilizó la definición que brinda la Organización Mundial de la Salud (OMS), considerando a toda persona desde el nacimiento hasta los 18 años ^11^.

El HEP es reconocido desde el año 2005 como categoría III-1 y es uno de los principales centros especializados en el manejo de emergencias y urgencias pediátricas, como la extracción de cuerpo extraño en la vía aérea ^12^.

### Criterios de inclusión

Se consideró a todos aquellos pacientes entre 0 y 18 años, que tuvieron como diagnóstico de alta un código de clasificación internacional de enfermedades 10ª (CIE-10) T17 (cuerpo extraño en vía aérea), sometidos a broncoscopia (rígida o flexible) y que fueron atendidos entre 2014 y 2019 en el HEP.

### Criterios de exclusión

Se excluyeron aquellos pacientes con una localización del cuerpo extraño distinta a la laringe, tráquea o árbol bronquial derecho o izquierdo y/o aquellos en los que el tiempo de enfermedad fue mayor de 18 días, considerando que tiempos mayores no serían emergencias pediátricas ^13^. Los pacientes con ausencia de cuerpo extraño y las historias clínicas que no cumplieron con un llenado adecuado también fueron excluidas.

### Cálculo de tamaño de muestra

Para el cálculo de tamaño de muestra se utilizó el artículo realizado por Tomaske *et al.*^6^. El estudio mencionado encontró que, los pacientes que tuvieron un tiempo de enfermedad mayor a 24 horas (expuestos) presentaban un riesgo de alta tardía del 40% y el riesgo de los que tuvieron un tiempo de enfermedad menor a 24 horas (no expuestos) fue del 29,1%. Se consideró la razón de no expuestos/expuestos de 0,60. Con estos valores se realizó el cálculo de tamaño muestral en el programa EPIDAT ® versión 4.2 (Xunta de Galicia, GAL, ESP), obteniéndose un tamaño de muestra inicial de 183 pacientes.

### Recolección de datos

Los datos fueron obtenidos mediante la revisión de historias clínicas de pacientes pediátricos del HEP. La recolección de datos se realizó solicitando un permiso al área de Investigación y Docencia del HEP. A fin de identificar a todos los pacientes con diagnóstico de cuerpo extraño en vías aéreas, se solicitó todas las historias que incluían pacientes con diagnóstico, manejo y alta del paciente. La recolección de datos fue realizada por dos autores (EAHJ y AKL) mediante una ficha de recolección de datos, donde se extrajeron todas las variables incluidas en el estudio.

### Variables

Respecto a la definición de las variables, se consideró como variable independiente al tiempo de enfermedad previo a la broncoscopia, definida como el tiempo transcurrido desde la aspiración del cuerpo extraño. Esta variable se categorizó en dos grupos (0 a≤2 días y >2 días) ^14^. La variable dependiente fue el tiempo de alta posbroncoscopia, definida como el tiempo transcurrido desde la extracción del cuerpo extraño hasta el alta del paciente, y fue categorizada en alta temprana, cuando ocurrió antes o a las 24 horas y como alta tardía, cuando ocurrió después de las 24 horas ^15^.

Dentro de las covariables consideramos características sociodemográficas como el sexo (masculino o femenino), la edad (años), procedencia (Lima o provincia), hospitalización previa y el tipo de paciente (directos o referidos). Además, se consideraron los hallazgos clínicos (tos, sibilancias, disminución del murmullo vesicular, hallazgos anormales en la radiografía de tórax), y maniobras de rescate fuera del establecimiento de salud (barrido de boca, maniobra de Heimlich y traqueotomía) ^10^. Dentro de las características de la broncoscopía, se consideró la localización del cuerpo extraño (tráquea, árbol bronquial derecho, árbol bronquial izquierdo o laringe) ^6^, el tipo de cuerpo extraño (orgánico e inorgánico) ^10^, el tipo de broncoscopía realizada de diagnóstico (rígida, flexible o ambas) y de extracción (rígida o flexible), al igual que la duración de broncoscopía en minutos (numérica) y la presencia de complicaciones (neumonía, atelectasia, neumotórax) ^10^.

### Análisis Estadístico

Para el análisis estadístico, se elaboró una base de datos mediante doble digitación en el programa Microsoft Excel 2019 ® (Microsoft Corporation, CA, USA) y exportado al paquete estadístico STATA 16 ® (Stata Corporation, College Station, Texas, USA). Se realizó la depuración de la base de datos y se eliminaron las historias clínicas incompletas para las variables de interés principales y secundarias.

Para el análisis descriptivo, las variables numéricas se evaluaron a través de la prueba de Shapiro Wilk para verificar si presentaban o no una distribución normal. De esta manera, se procedió a describir las variables numéricas: edad y duración de la broncoscopia de extracción mediante medianas y rangos intercuartílicos (RIQ) debido a que ninguna de las dos variables presentó una distribución normal. Para la descripción de las variables categóricas se utilizaron frecuencias y porcentajes. En el análisis bivariado, se utilizó la prueba exacta de Fisher para determinar la diferencia entre las proporciones de las variables categóricas y la variable dependiente, mientras que para las variables numéricas se utilizó la prueba U de Mann-Whitney. Se consideró un p<0,05 como estadísticamente significativo.

Finalmente, en el análisis multivariado, se utilizó el modelo de regresión de Poisson para calcular el riesgo relativo (RR) y sus respectivos intervalos de confianza (IC) al 95%. Se calculó un modelo ajustado por las siguientes variables: edad, género, tipo de cuerpo extraño y barrido de la boca con el dedo como maniobra de rescate. Esta última variable fue incluida por criterio estadístico. El tipo de cuerpo extraño se consideró como posible variable confusora a pesar de no obtener un valor estadísticamente significativo en nuestro estudio, en virtud de su criterio epidemiológico ^16^.

### Aspectos éticos

El protocolo de estudio se presentó al Comité de ética de la Universidad Peruana de Ciencias Aplicadas y la Oficina de Apoyo a la Docencia e Investigación del Hospital de Emergencias Pediátricas (código FSC-CEI/55-10-20), y solo se realizó la recolección de datos una vez obtenida la aprobación de ambas entidades. No se realizaron intervenciones sobre los sujetos de estudio, ni se incluyeron los datos de identificación personal de los pacientes. Los datos clínicos recolectados para nuestro estudio fueron ingresados a un banco de datos, donde solo los investigadores principales tuvieron acceso.

## RESULTADOS

Se revisaron un total de 324 historias clínicas de pacientes pediátricos con diagnóstico de cuerpo extraño en vías aéreas. Estos pacientes fueron atendidos para extracción con el uso de fibrobroncoscopia y/o broncoscopio rígido en el HEP de la ciudad de Lima, Perú. De estas 324 historias, 141 fueron excluidas y 183 historias fueron incluidas en el estudio (Figura 1).

**Figura 1:** Flujograma de la selección de los participantes

Del total de pacientes pediátricos incluidos en el estudio, se encontró que según sus características sociodemográficas, 120 (65,6%) pacientes fueron hombres, con una mediana de edad de 2,4 años (RIQ: 1,3-7). La mayoría de los pacientes (80,3%) llegaron al HEP por referencia (Tabla 1). En cuanto a las variables clínicas y de manejo del paciente con diagnóstico de cuerpo extraño, el síntoma más frecuente fue la tos (73,2%). Finalmente, la maniobra de rescate, previa a la extracción más utilizada fue el barrido de boca con el dedo (37,2%) (Tabla 1).

**Tabla 1:** Características sociodemográficas, clínicas y de intervención previa al manejo y tratamiento de los pacientes pediátricos con diagnóstico de cuerpo extraño en vías aéreas (n=183 pacientes).

Características	n	%
Género		
Masculino	120	65,6
Femenino	63	34,4
Edad (en años)^a^	2,4	1,3-7
Procedencia		
Lima	65	35,5
Provincia	118	64,5
Tipo de paciente		
Directo	36	19,7
Referido	147	80,3
Hospitalización previa		
Sí	33	18,0
No	150	82,0
Hallazgos clínicos		
Tos		
Sí	134	73,2
No	49	26,8
Sibilancias		
Sí	53	29,0
No	130	71,0
Disminución de murmullo vesicular		
Sí	99	54,1
No	84	45,9
Radiografía de tórax		
Normal	66	35,7
Anormal	117	64,3
Maniobras de rescate fuera del establecimiento		
Barrido de boca		
Sí	68	37,2
No	115	62,8
Maniobra de Heimlich		
Sí	5	2,7
No	178	97,3
Traqueotomía		
Si	1	0,6
No	182	99,4

a Mediana y rango intercuartílico.

Según las características de la broncoscopia realizada a los pacientes pediátricos con diagnóstico de cuerpo extraño en vías aéreas, se encontró que la mayoría de los pacientes tuvo un tiempo de enfermedad previo a broncoscopia menor o igual a dos días (56,8%). Asimismo, la localización más frecuente del cuerpo extraño fue en el árbol bronquial derecho (46,6%). El tipo de broncoscopia más frecuente fue la broncoscopia flexible, como técnica inicial (53,0%)y como procedimiento que realizó la extracción del cuerpo extraño (54,1%). La duración de la broncoscopia tuvo una mediana de 20 minutos (RIQ: 15-30) (Tabla 2).

**Tabla 2:** Características de la broncoscopía de los pacientes pediátricos (n=183 pacientes).

Características	n	%
Tiempo de enfermedad previo a la broncoscopia		
0 a 2 días	104	56,8
> 2 días	79	43,2
Localización del cuerpo extraño		
Tráquea	27	14,8
Árbol bronquial derecho	78	42,6
Arbol bronquial izquierdo	52	28,4
Laringe	26	14,2
Broncoscopia realizada		
Broncoscopio rígido	8	4,4
Broncoscopio flexible	97	53,0
Ambas	78	42,6
Broncoscopio de extracción		
Broncoscopio rígido	84	45,9
Broncoscopio flexible	99	54,1
Duración de la broncospia (en minutos)^a^	20	15-30

a Mediana y rango intercuartílico.

Respecto a las características de los pacientes pediátricos posextracción del cuerpo extraño, la mayoría de los pacientes (72,7%) tuvo un alta temprana (antes o igual a las 24 horas). El tipo de cuerpo extraño predominante en los pacientes pediátricos fue de material orgánico (59,6%). En cuanto a las complicaciones, la más frecuente fue la neumonía (24,6%), seguida de atelectasia (18,0%) y neumotórax (1,6%), siendo esta la complicación menos frecuente (Tabla 3).

**Tabla 3:** Características de los pacientes pediátricos posextracción del cuerpo extraño (n=183 pacientes).

Características	n	%
Tiempo de alta posbroncoscopia		
Alta temprana (≤24 horas)	133	72,7
Alta tardía (>24 horas)	50	27,3
Tipo de cuerpo extraño		
Orgánico	109	59,6
Inorgánico	74	40,4
Complicaciones		
Neumonía		
Sí	45	24,6
No	138	75,4
Atelectasia		
Sí	33	18,0
No	150	82,0
Neumotórax		
Sí	3	1,6
No	180	98,4

Respecto al análisis bivariado, no se encontró diferencias entre la mediana de la edad del paciente y el tiempo de hospitalización posextracción. Asimismo, no hubo diferencias entre las proporciones de la hospitalización previa de pacientes con el tiempo de hospitalización posextracción. Sin embargo, se encontraron diferencias entre las proporciones del barrido de la boca con el dedo como maniobra de rescate y el tiempo de hospitalización posextracción (Tabla 4). Adicionalmente, se encontraron diferencias entre las proporciones del tiempo de enfermedad previo a broncoscopia y el tiempo de alta posbroncoscopia (p<0,001). Finalmente, se hallaron diferencias entre las proporciones de la ocurrencia de neumonía y el tiempo de hospitalización posextracción (p<0,001) (Tabla 5).

**Tabla 4:** Contraste de variables sociodemográficas y clínicas con el tiempo de permanencia del paciente en el centro de salud posextracción (n=183 pacientes).

Características	Alta temprana (≤24 horas) (n = 133)		Alta tardía (>24 horas) (n = 50)		Valor de p
	n	%	n	%	
Género					
Masculino	90	75,0	30	25,0	0,384
Femenino	43	68,3	20	31,7	
Edad (en años)^a^	2,6	1,3-7,3	2,1	1,1-5,8	0,931^b^
Procedencia					
Lima	53	81,5	12	18,5	0,057
Provincia	80	67,8	38	32,2	
Tipo de paciente					
Directo	28	77,8	8	22,2	0,534
Referido	105	71,4	42	28,6	
Hospitalización previa					
Sí	20	60,6	13	39,4	0,129
No	113	75,3	37	24,7	
Hallazgos clínicos					
Tos					
Sí	96	71,6	38	28,4	0,709
No	37	75,5	12	24,5	
Sibilancias					
Sí	38	71,7	15	28,3	0,856
No	95	73,1	35	26,9	
Disminución de murmullo vesicular					
Sí	67	67,7	32	32,3	0,134
No	66	78,6	18	21,4	
Radiografía de tórax					
Normal	54	83,1	11	16,9	0,024
Anormal	78	66,7	39	33,3	
Maniobras de rescate fuera del establecimiento					
Barrido de boca					
Sí	56	82,0	12	17,7	0,026
No	77	67,0	38	33,0	
Maniobra de Heimlich					
Sí	2	40,0	3	60,0	0,127
No	131	73,6	47	26,4	
Traqueotomía					
Si	0	0,0	1	100,0	0,273
No	133	73,1	49	26,9	

a Mediana y rango intercuartílico; ^b^ valor de p calculado mediante la prueba U de Mann-Whitney.

**Tabla 5:** Contraste de variables de la broncoscopía y complicaciones del paciente pediátrico con el tiempo de estancia hospitalaria posextracción del cuerpo extraño (n=183 pacientes).

Características	Alta temprana (≤24 horas) (n = 133)		Alta tardía (>24 horas) (n = 50)		Valor de p
	n	%	n	%	
Tiempo de enfermedad previo a la broncoscopia					
0 a 2 días	84	85,6	15	14,4	0,001
> 2 días	44	55,7	35	44,3	
Localización del cuerpo extraño					
Tráquea	21	77,8	6	22,2	0,941
Árbol bronquial derecho	56	71,8	22	28,2	
Árbol bronquial izquierdo	37	71,2	15	28,9	
Laringe	19	73,1	7	26,9	
Tipo de cuerpo extraño					
Orgánico	77	70,6	32	29,4	0,502
Inorgánico	56	75,7	18	24,3	
Broncoscopia realizada					
Broncoscopio rígido	8	100,0	0	0,0	0,124
Broncoscopio flexible	72	74,2	25	25,8	
Ambas	53	68,0	25	32,0	
Broncoscopio de extracción					
Broncoscopio rígido	59	70,2	25	29,8	0,510
Broncoscopio flexible	74	74,8	25	25,2	
Duración de la broncoscopia (en minutos)^a^	20	15-30	25	15-35	0,169 b
Complicaciones					
Neumonía					
Sí	13	28,9	32	71,1	0,001
No	120	87,0	18	12,0	
Atelectasia					
Sí	19	57,6	14	42,4	0,050
No	114	76,0	38	24,0	
Neumotórax					
Sí	2	66,7	1	33,3	1,000
No	131	72,8	49	27,3	

a Mediana y rango intercuartílico; ^b^ valor de p calculado mediante la prueba U de Mann-Whitney.

En el análisis multivariado, el modelo ajustado por las variables edad, sexo, tipo de cuerpo extraño y técnica de barrido de boca mostró en aquellos pacientes con más de 48 horas de tiempo de enfermedad previo a la broncoscopia de extracción, el riesgo de tener alta tardía aumenta en 2,94 veces (IC 95%: 1,72-5,01; p=0,001) (Tabla 6).

**Tabla 6:** Análisis de regresión crudo y ajustado para el tiempo de hospitalización mayor de 24 horas posextracción del cuerpo extraño en el paciente pediátrico en el Hospital de Emergencias Pediátricas (n=183 pacientes).

Características	Análisis crudo		Análisis ajustado^a^	
	RR (IC 95%)	Valor de p	RR (IC 95%)	Valor de p
Tiempo de enfermedad previo a la broncoscopia				
0 a 2 días	Ref.		Ref.	
>2 días	3,07 (1,81-5,22)	0,001	2,94 (1,72-5,01)	0,001

RR: riesgo relativo, IC: intervalo de confianza

a Ajustado por edad, género, tipo de cuerpo extraño y barrido de boca.

## DISCUSIÓN

De los 183 pacientes incluidos, la localización más frecuente fue en el árbol bronquial derecho y el tipo de cuerpo extraño más frecuente fue de materiales orgánicos. El 72,7% de pacientes tuvieron un alta antes de las 24 horas. Como hallazgo principal, se encontró una asociación el tiempo de enfermedad previo a la broncoscopia mayor a dos días y el alta tardía.

El momento de aspiración ocurre esencialmente al estar comiendo o jugando, usualmente bajo la supervisión de un adulto ^17^, lo que corresponde a la alta exposición a nuevos estímulos y alimentos en edades tempranas, siendo los objetos orgánicos los más frecuentes ^18^, especialmente, los alimentos ^19^. Este hallazgo es consistente con otro estudio realizado por Bing Zhong *et al*. ^16^. Particularmente, en nuestro estudio, el 59,6% de los cuerpos extraños que se extrajeron fueron de tipo orgánico. Este hallazgo es relevante al considerar que el tipo de cuerpo extraño está asociado con el tiempo de estancia hospitalaria ^8^, y que distintos tipos de cuerpos extraños pueden asociarse a distintos tipos de lesiones, por ejemplo, los cuerpos orgánicos se asocian a injuria en vías respiratorias superiores ^20^.

Nuestro estudio muestra una asociación entre el tiempo de enfermedad previo a la broncoscopia y el tiempo de hospitalización luego de la extracción de cuerpo extraño. Sin embargo, el estudio de Hidaka *et al.*^8^, no encontró asociación entre las variables de interés, donde describen que el 74% de los casos se sometieron a extracción en los primeros dos días desde la aspiración del cuerpo extraño, mientras que en nuestro estudio tan solo el 53,8% de los casos se sometieron a extracción en los primeros dos días desde la aspiración del cuerpo extraño. Esta diferencia indicaría que en otros contextos como Japón, el diagnóstico y extracción del cuerpo extraño, se realiza en menor tiempo a comparación de nuestro medio, disminuyendo así el riesgo de complicaciones y tiempo de hospitalización. Así, nuestro hallazgo podría ser explicado por la problemática del sistema de referencias médicas en el Perú y disponibilidad de especialistas ^10^. Asimismo, el estudio realizado por Hidaka *et al.* describe una baja prevalencia de aspiración de cuerpo extraño en Japón ^8^, pues durante los 23 años que evaluaron (desde 1988 hasta el 2011), solo encontraron 77 casos de aspiración de cuerpo extraño. En contraste, nuestro estudio consideró un intervalo de tiempo de tan solo seis años (desde 2014 hasta 2019), encontrándose más de 183 casos. Por lo tanto, estas diferencias podrían conllevar a resultados disímiles entre ambos estudios.

No obstante, diversos estudios encontraron una asociación entre el tiempo de enfermedad y el tiempo de hospitalización postextracción ^7^^,^^21^. Por ejemplo, en un estudio realizado en China ^7^, se encontró una correlación entre el tiempo de retención de cuerpo extraño en la vía aérea y el tiempo de hospitalización. A pesar de que esta correlación fue baja (r=0,189), fue estadísticamente significativa (p<0,001). En nuestro estudio, el 56,8% tuvo un tiempo de enfermedad previo a la broncoscopia menor o igual a dos días, resultado similar al reportado en el estudio realizado en población china, donde el 68,5% tuvo un tiempo de tres días o menos, lo que generaría que ambos estudios encontraran una relación entre ambas variables. Sin embargo, para los autores del estudio realizado en China, no fue posible plantear un punto de corte para el tiempo de enfermedad previo a la broncoscopia, pues consideraron esta variable como numérica. Adicionalmente, otro estudio realizado en población pediátrica española mostró un resultado similar al nuestro, donde los autores reportan que hubo una asociación significativa entre el retraso diagnóstico y la aparición de complicaciones a largo plazo, sin abordar acontecimientos adversos intraoperatorios ^21^.

Un tiempo prolongado de enfermedad previo a la broncoscopia de extracción duplica el riesgo de hospitalización posextracción. Aunque la asfixia y el paro cardiorrespiratorio son más frecuentes poco después de la aspiración, también puede ocurrir en días posteriores ^22^. Un diagnóstico tardío se asocia frecuentemente a atelectasias, abscesos pulmonares y una mayor morbimortalidad ^22^^-^^24^, debido a la inflamación que ocurre a nivel de las vías aéreas inferiores ^16^. De hecho, la literatura médica describe que, al aspirar un cuerpo extraño, se produce inflamación en la vía aérea, la cual, prolonga el tiempo de recuperación pulmonar ^25^. La importancia de este hallazgo se observa en un estudio realizado por Man Ki Chung *et al*., donde describen una asociación entre los hallazgos inflamatorios de la vía aérea previo a la extracción de un cuerpo extraño y un retraso en la recuperación pulmonar ^25^. Este hecho puede generar una necesidad de hospitalización prolongada, lo que a su vez, genera mayor riesgo de adquirir infecciones nosocomiales ^26^.

El retraso diagnóstico o terapéutico puede explicarse por causas asociadas al médico, recursos, padres o historia clínica poco informativa ^20^. Respecto a la historia clínica, el cuadro puede presentarse, incluso sin el antecedente de ahogo o asfixia en población pediátrica ^27^. Por este motivo, nuestros resultados resaltan la importancia del manejo oportuno y correcta evaluación de los pacientes dentro de las primeras 48 horas. De forma complementaria, a fin de asegurar el manejo oportuno y mejorar el pronóstico, se recomienda capacitar a los cuidadores del niño, agilizar los sistemas de referencia y fortalecer programas sobre el uso de broncoscopía rígida y flexible en pacientes pediátricos. Por lo tanto, en consonancia con nuestros hallazgos, se debe priorizar la disponibilidad de profesionales capacitados y recursos suficientes en centros de salud para el manejo oportuno de pacientes pediátricos con aspiración de cuerpo extraño, lo que permitiría reducir complicaciones y mejorar el pronóstico de estos pacientes.

Este estudio presenta limitaciones. No se evaluó la evolución del paciente una vez que se le dio de alta del HEP debido a la naturaleza del diseño del del estudio. A pesar de que solo evaluamos los datos que se encontraban descritos en las historias clínicas, las complicaciones posextracción son infrecuentes ^18^^,^^21^^,^^27^ ^28^ y en caso de haberlas presentado, el paciente hubiera optado por retornar. Debido a que los datos fueron recolectados de forma manual mediante una ficha de recolección de datos, podría existir un sesgo de información. Además, no se incluyeron otras variables relevantes para realizar este estudio, como la actividad que se encontraba realizando el paciente al momento de la aspiración, ya que es un dato que se registra en la historia clínica con baja frecuencia. Con respecto a la validez externa, debido a que los datos fueron obtenidos de un único hospital, los datos no pueden ser extrapolados a poblaciones distintas. Sin embargo, se debe considerar que el HEP es una de las principales instituciones de referencia de pacientes pediátricos en Lima, Perú, lo que permite determinar la asociación de interés de manera exploratoria. Finalmente, no existe una estandarización de las principales variables (tiempo de alta y tiempo de enfermedad previo a la broncoscopía). No obstante, nuestra investigación utiliza puntos de corte descritos en estudios similares ^14^^,^^15^.

De acuerdo con nuestros resultados, los niños menores de tres años y de sexo masculino representan la mayoría de los casos de aspiración de cuerpo extraño. Además, aquellos pacientes con un tiempo de enfermedad mayor a dos días entre la aspiración de cuerpo extraño y la broncoscopia tuvieron 2.94 más riesgo de una estancia hospitalaria prolongada (más de 24 horas) al ajustar por edad, sexo, tipo de cuerpo extraño y barrido de boca como maniobra de rescate.
